# Messy but worth it: human-centred design as applied within a successful vaccine-promotive campaign

**DOI:** 10.1136/bmjgh-2023-014870

**Published:** 2024-07-29

**Authors:** Mark Donald C Reñosa, Kate Bärnighausen, Jonas Wachinger, Vivienne Endoma, Jeniffer Landicho, Mila F Aligato, Jhoys Landicho-Guevarra, Thea Andrea Bravo, Kerry Scott, Till Bärnighausen, Shannon A McMahon

**Affiliations:** 1 Heidelberg Institute of Global Health, Ruprecht-Karls-Universität Heidelberg, Heidelberg, Germany; 2 Department of Epidemiology and Biostatistics, Research Institute for Tropical Medicine - Department of Health, Muntinlupa City, Metro Manila, Philippines; 3 School of Global Health, Faculty of Health, York University, Toronto, Ontario, Canada; 4 International Health Department, Johns Hopkins University Bloomberg School of Public Health, Baltimore, Maryland, USA

**Keywords:** Global Health, Vaccines, Health policy, Immunisation, Other study design

## Abstract

Human-centred design (HCD) is an approach to problem-solving that prioritises understanding and meeting the needs of the end-users. Researchers and designers practice empathic listening as users share their perspectives, thereby enabling a variety of stakeholders to cocreate effective solutions. While a valuable and, in theory, straightforward process, HCD in practice can be chaotic: Practitioners often struggle to navigate an excess of (often conflicting) ideas and to strike a balance between problem-understanding and problem-solving. In this practice paper, we outline our own experiences with HCD, which ultimately resulted in the development of a successful video-based intervention to bolster vaccine confidence in the Philippines. We highlight the use of ‘radical circles’ to overcome roadblocks and navigate tensions. Radical circles entail groups of individuals with divergent opinions and identities engaging in critical analysis of a given idea, actively challenging standard ways of thinking, and ultimately, generating solutions. Employing radical circles enabled us to innovate and adapt to new perspectives that emerged along the non-linear HCD pathway. Our incorporation of radical circles into HCD methodology demonstrates its potential as a powerful complementary step in the meaning-making process. In our view, radical circles could enrich HCD processes and provide a solution to design overcrowding, leading to meaningful, transformative and successful interventions.

**Summary box**

- Researchers in the field of global health are increasingly highlighting the complexity, messiness and time-consuming nature of human-centred design (HCD).
- While messy, HCD’s emphasis on capturing perspectives from end-users remains exceptionally valuable in the health field, which is critiqued for being insular and hierarchical in nature.
- Our own research began by following a traditional HCD approach (capturing perspectives from end-users to inspire and ideate solutions), but we balanced this approach with ‘radical circles’—convening individuals who could provide critical feedback and codevelop solutions to idea overcrowding—and found that such circles can assist in managing complexities encountered in design research.
- Drawing from our experience, careful curation and close engagement within radical circles throughout revision and iteration can support product creation and help to develop a meaningful and successful intervention.

## Introduction

 Human-centred design (HCD), which champions the capture of end-users’ priorities, perspectives and needs, has been increasingly used in global health research and practice.^1^ The HCD approach emphasises conceptualising problems and considering solutions in partnership with a variety of stakeholders including, perhaps most prominently, end-users or those for whom a product, service or innovation is developed.^2^ In terms of problem conceptualisation and intervention development, HCD urges sincere engagement with end-users to see the world through their eyes and to then ideate challenges and solutions collectively.^1^,^3^ Collaborating with a variety of stakeholders refers to drawing insights not only from end-users and the ‘usual suspects’ engaged in intervention research (eg, patients, health practitioners, community stakeholders, fellow researchers or academics), but also collecting perspectives from individuals whose talent may not be as well recognised within global health such as artists, actors, engineers or software programmers to name a few.^1 2^

While in theory, the HCD approach sounds straightforward, in practice it is often complex, messy and time-consuming.^4 5^ Academic teams tend to exhibit a preference for maintaining the status quo of health intervention development, likely due to scarcity in terms of finances and time, which restricts capacity to undertake additional design work. This preference primarily manifests in two approaches that both lack a design component: (1) taking solutions or messages developed by scientists and attempting to roll them out directly or (2) taking ideas generated by communities or organisations in one location and attempting to transplant or scale them up. Both approaches tend to exclude designers and overlook end-users. On the other hand, teams that favour design components are often more conversant with formative research methodologies,^6^ which reflect HCD but are slightly less time-consuming and more focused on the form and function of a particular intervention. Among scholars and practitioners engaged in HCD, several have described HCD as a fluid, iterative and creative process that can generate unpredictable outcomes and requires multiple rounds of testing.^7^,^9^ The process, therefore, is at odds with more typical processes for addressing issues related to social behaviour in public health, which are constrained by predetermined implementation plans, budgets and timelines. At present, there are few discussions on how to contend with challenges that may emerge when reconciling the discrete and tidy nature of the typical scientific process with the fluid and iterative process that aligns with the spirit of artistic creation which is inherent to HCD.

Our team recently drew on HCD to develop a vaccine-promotive campaign that proved successful in a randomised controlled trial.^10 11^ When presenting or discussing our trial,^11^,^13^ we have consistently been asked to outline our HCD process; privately, we have also been asked whether we too found the design process ‘confusing,’ ‘overwhelming’ or ‘slow’. In this practice paper, we outline our HCD process (including the main phases we followed), and we describe one key workaround that our team employed (ie, radical circles^14^), which helped us to overcome the complexities and iterative tensions inherent to HCD. Lastly, we discuss methodological and practical modifications as well as lessons learnt to help other intervention designers who are navigating the challenges of HCD, or who may wish to pursue an HCD approach in the future.

### Project SALUBONG: building vaccine confidence in the Philippines

Project SALUBONG sought to gain a better understanding of the current state of vaccine hesitancy in rural and urban areas in the Philippines.^10^ Vaccine confidence has declined in the Philippines following a ‘Dengvaxia’ controversy in 2017 (Dengvaxia is a dengue vaccine developed and produced by Sanofi Pasteur).^15^ The decline in vaccine confidence and uptick in vaccine refusal led to the country losing its 19-year polio-free status and sparked measles outbreaks across several islands in 2019.^15 16^ Our project aimed to codevelop an intervention to bolster vaccine confidence by using local narratives, designing and refining an HCD-driven intervention and working with end-users and stakeholders (policymakers, community leaders, health promotion and communication specialists).^10^ In our work, we define end-users as individuals receiving the service (caregivers and their families) as well as those delivering vaccinations (healthcare workers).

Following the principles of HCD, our project employed an applied, mixed-methods approach over four years beginning in 2020.^10^ However, due to the COVID-19 pandemic, we were forced to forgo in-person data collection. We, therefore, transitioned all data collection procedures to remote qualitative^17 18^ and quantitative^11^ approaches in line with the procedures outlined in a published protocol.^10^ All data collection activities were performed in Filipino. Qualitative data were audio recorded or video recorded, transcribed verbatim and translated into English. All data collection activities were performed and led by Filipino researchers (VE, JL, MA, JL-G and TAB) including the lead author (MDCR). Transcription and translation of transcripts were performed by trained data transcribers who are fluent in Filipino and English. Once translation was completed, the team quality checked content and translation accuracy in at least half of all transcripts. Further methodological details of our project are available elsewhere.^10 17 18^ For more details on the background of coauthors and composition of the research team, please see the author reflexivity included as online supplemental file.

In the spirit of HCD, we did not initially know what our intervention would entail. Our goal was to develop a promotive message to inform families about vaccines in a manner that resonated with their values, context and household dynamics, but the programme’s phrasing and narrative (if any) remained open. In sum, this approach was much broader compared with our previous health interventions in that we did not have an a priori decision in terms of the intervention’s format. In our HCD approach, we aligned with the general methodological flow starting with empathising, followed by defining and ideating, then prototyping, and finally testing.

### Step 1: empathise

In the wake of the Dengvaxia vaccine scare of 2017^19^ and the COVID-19 pandemic in the Philippines, we first conducted remote in-depth interviews (IDIs) with Filipino policymakers (n=19) to understand general challenges for vaccination campaigns.^20^ Our findings implied that widespread vaccination panic fosters public scepticism and is exacerbated by contextual and political influences that put pressure not just on vaccine confidence but also on health programmes more generally.^20^

We then performed remote IDIs among vaccine hesitant (n=44)^21 22^ and vaccine accepting (n=11) caregivers, yielding salient stories that served as the intervention’s backbone during the predevelopment phase. Along with discussing vaccines in general, we also investigated respondents’ preferred vaccine information delivery channels, including their chosen medium, platforms and trusted messengers. Among the most salient themes across respondent groups was a desire for personal stories coupled with a disinterest and have minimal interest in scientific insights about vaccines beyond side effects.^21^

**Figure 1:**
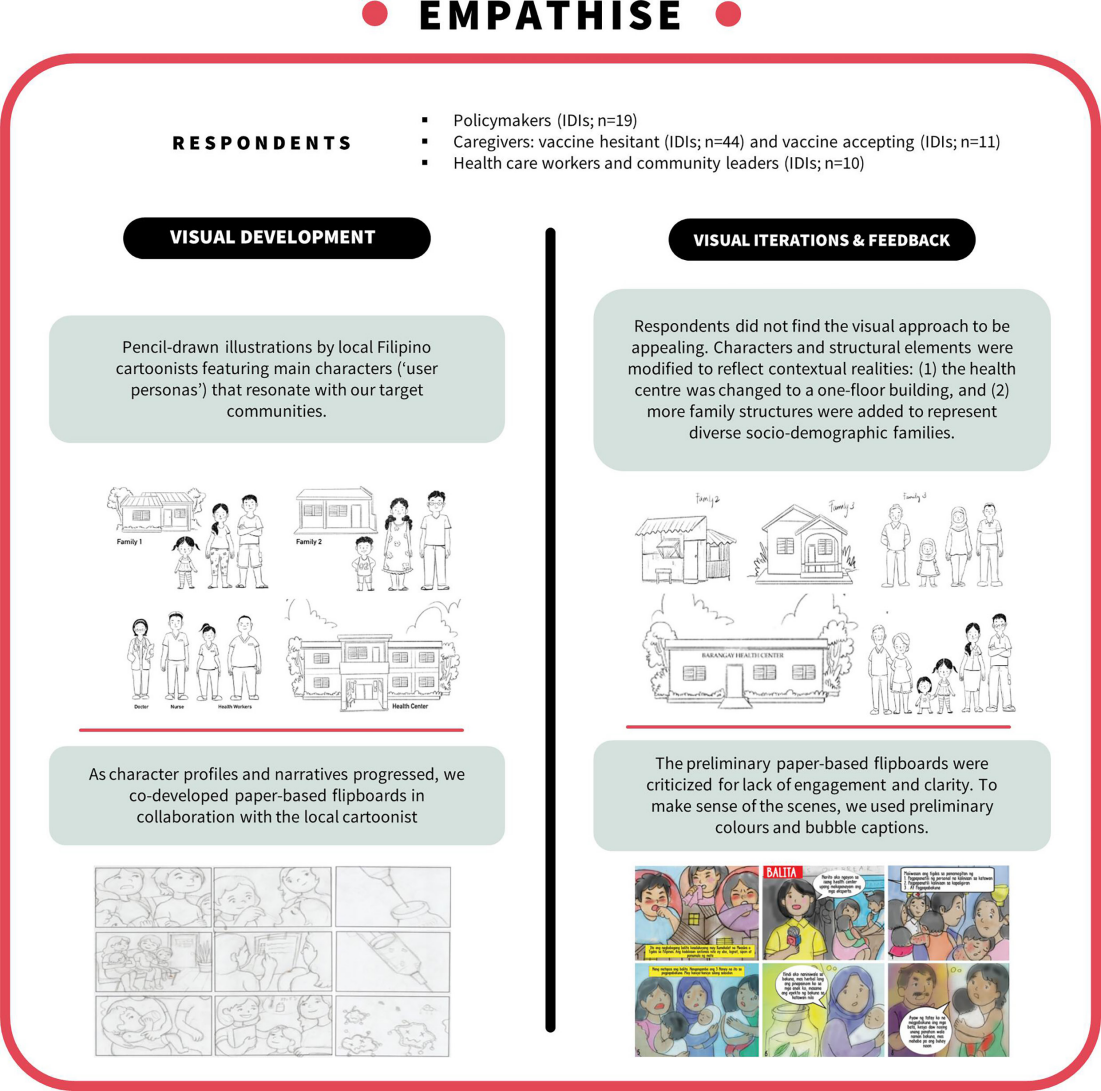
Empathise phase: SALUBONG storyboard progression. IDIs, in-depth interviews.

Regarding intervention delivery, respondents described an openness to learning more about vaccines while waiting in health centres or when passing time in general. In terms of delivery agents, respondents only described interest in learning about vaccines from sources they considered credible (medical doctors, nurses and other health professionals). However, they wanted these individuals to be more forthright, motivating and open when discussing vaccination.^21^ We, thus, decided to develop a storyboard (ie, a sequence of illustrations that present a narrative) that depicted end-users discussing vaccines with healthcare workers, and that could potentially be printed as educational pamphlets or converted into radio scripts or short animated advertisements, depending on user preferences (see figure 1).

At the midpoint of interviewing, we partnered with a graphical design firm and began creating user personas (ie, fictitious profiles that include individuals’ short biography, age, gender, etc),^23^ choosing narratives that reflected reality and could resonate with our target group’s concerns. These preliminary profile sketches were presented and iterated in roughly half of caregiver interviews. Feedback primarily pertained to respondents’ sentiments towards the personas, by asking questions such as: Does this character reflect their struggles with vaccines? Does this character appear genuine?

As the profiles and narratives progressed, we transformed them into full storyboards^23 24^ that were placed in a sequence on paper-based flipboards. Flipboards in hand, we performed think-aloud exercise with both caregivers and two new study groups (healthcare workers (medical doctors, nurses and midwives; n=7) and community leaders (n=3)); we showed the flipboards in video calls, and/or screen shared digital photos of the paper-based storyboards. We had originally planned to have local cartoonists and designers join these online interviews as observers, or as individuals who could modify character features instantaneously. We ultimately decided to forgo this approach because of the sensitivity of the issue (vaccines) and ethical concerns (privacy). Local cartoonists were thus debriefed after interviews and asked to iterate their designs based on respondent feedback. By the end of the empathise phase, we had a clear sense of which messages to include (or exclude) in our intervention, and which messenger types to draw from (or avoid). We remained uncertain of the format of the intervention (booklet, pamphlet and digital video).

### Steps 2–3: define and ideate

Our define and ideate phase overlapped with our empathise phase which ensured a continuous cycle of understanding, ideation and refinement of our codesigned intervention. By embracing the non-linear and iterative nature of HCD, our goal was to achieve a balanced fusion of divergent and convergent ideas, enabling us to capture a fuller context over time. Our iterative workflow guaranteed that our next step (prototype) was informed by a deeper understanding of end-users’ needs while simultaneously fostering creativity and exploration in the ideation process.

Our design and ideate phase entailed focus group discussions (FGDs) among caregivers who described themselves as either vaccine hesitant (1 FGD, n=5) or vaccine accepting (4 FGDs, n=22) and community health workers. We stratified the caregivers for FGDs according to their vaccination views and conducted a separate FGD with community health workers to prevent contamination, conflict and imbalances of power during discussions.^10^ During this phase, along with discussing socially held norms on vaccines and the public health system, we also gathered more feedback on the preliminary storyboards developed in the empathise phase.

Here, we learnt about the complementary and conflicting notions of responsibility of caregivers and healthcare workers that shape vaccine decision-making.^25^ We observed that caregivers are drawn into their responsibility of becoming a good parent, which included ensuring that their children are free from vaccine risks. Healthcare workers, on the other hand, were more focused on upholding their responsibility to maintain the health of the general community, which—in some cases—resulted in healthcare workers pressuring families to vaccinate. These opposing perspectives led to tensions within and across parties, with caregivers complaining about embarrassing experiences in the health centre^21^ and healthcare workers criticising caregivers for being too ‘stubborn’ or lazy to attend their scheduled vaccination.^25^

In terms of ideation, we moved beyond preliminary storyboards and worked on more intricate details of the stories to gauge, for example, which narratives most poignantly echoed vaccine sentiments and hopes. However, due to difficulties experienced with the online platform and because we felt we could not gather substantive, socially held attitudes or meaningful criticisms on the storyboards among caregivers in group settings,^18^ we shifted data collection from FGDs to IDIs. This change to remote IDIs allowed us to build a more intricate and thorough understanding of caregivers’ viewpoints, and our end-users’ needs. We undertook follow-up IDIs among previously interviewed vaccine hesitant caregivers who provided salient stories (n=3) to validate if the storyboards reflected their stories and narratives. We further expanded our data collection to include more IDIs of vaccine hesitant caregivers who had strong opinions regarding vaccines (refused vaccines, n=3) and who could provide a deeper understanding of their views and further iterate the storyboards. While the number of vaccine hesitant caregivers interviewed decreased in comparison to the empathise phase, this adjustment was made to enhance the quality and depth of insights gathered, rather than as a reflection of the importance attributed to engaging with this specific demographic group. Finally, we conducted additional FGDs among community health workers (4 FGDs, n=27) to understand community and health facility experiences and to gauge whether the storyboards resonated with health facility norms and practices.

**Figure 2:**
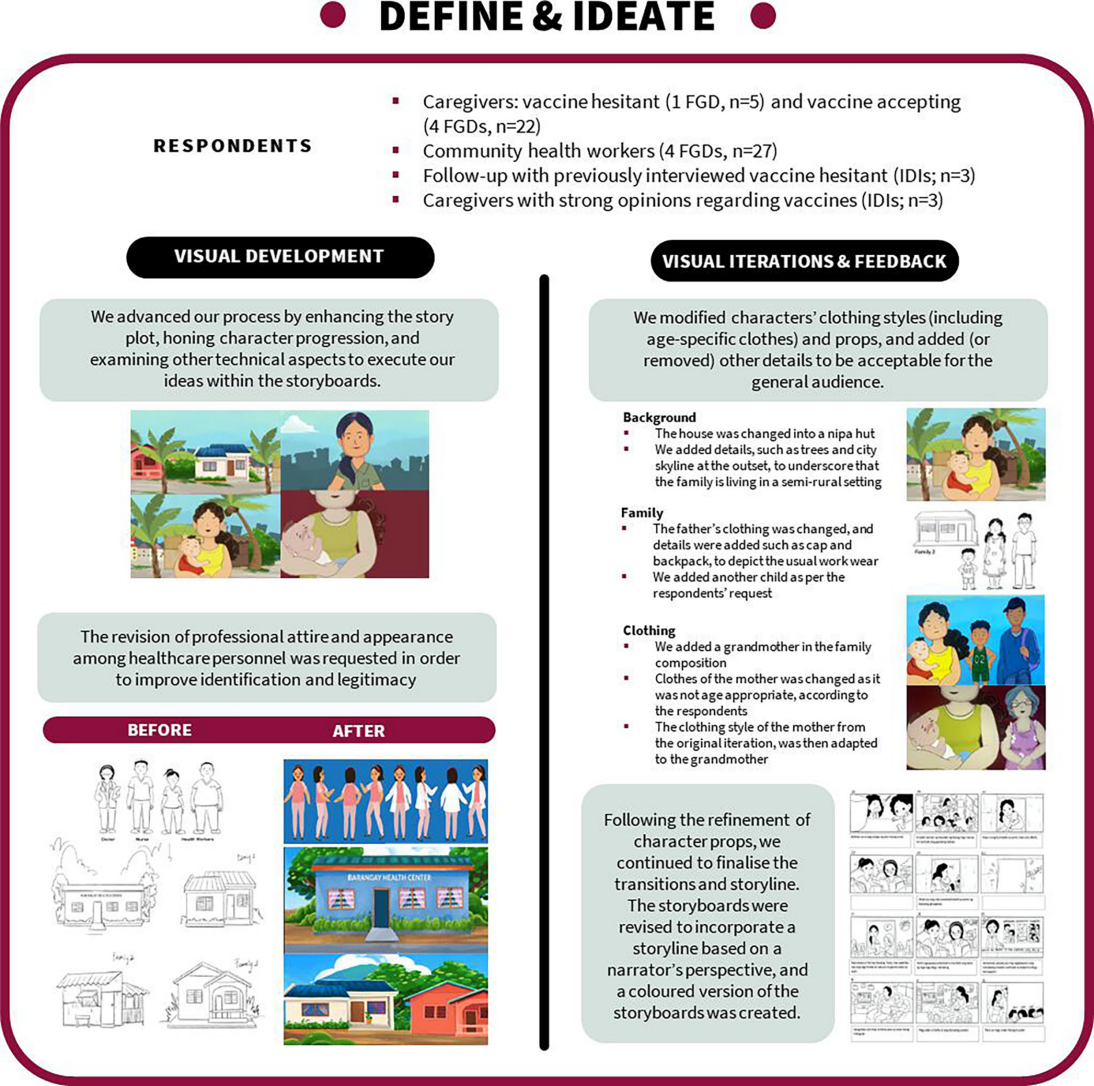
Define and ideate phase: SALUBONG storyboard progression. FGDs, focus group discussions; IDIs, in-depth interviews.

All respondent types highlighted a need for the storyboards to be tailored to the Filipino cultural context, and to feature characters of various ages, incomes, ethnicities and household compositions (eg, featuring families with different numbers of children). Respondents were also shown colour palettes and respondent groups consistently chose more vibrant colours schemes with higher contrast. We incorporated all feedback on character features and dresses, environment details, props and other technical aspects of the storyboards. At the end of this phase, we had developed a full-length series of paper-based storyboards that incorporated refined sketches of characters and context but were not yet generated into an animated format (see figure 2).

### Step 4: prototype

We performed IDIs with healthcare workers (midwives, n=14) and community health workers to ask for their feedback on the preliminary storyboards and visual analogues of the stories and to determine what, if anything, should be changed or added. These IDIs were used to direct us in terms of practicality (eg, paper vs video-based presentation) and preferences in terms of delivery style (spoken text to accompany the storyboards, one-on-one vs group delivery of the storyboards, stand-alone activity vs nested in current outreach).

Respondents discussed video-based formats as feasible and desirable. They highlighted an advantage of being able to display videos on televisions at health centres or during outreach events, as well as an option to distribute videos through social media platforms instead of relying on paper-based materials. Furthermore, the idea of paper-based formats was dropped due to concerns regarding sustainability and budgetary constraints. Healthcare workers described wanting a narrative style voice-over (eg, only one voice conveying the story plot) rather than dubbed character voices as they felt this would bolster clarity among the audience. Based on these inputs, our design team converted the storyboard narratives into a short-animated video. Respondents also mentioned the potential for implementing a stand-alone vaccination promotion initiative that could be integrated into broader child health programmes. We gained details about the mode, timing and geographic scope of intervention delivery, which aided in the strategic development of our intervention’s roll-out.

During this phase, we saw a significant influx of ideas, resulting in a high degree of ‘overcrowding’, characterised by the presence of unclear feedback, conflicting ideas and viewpoints, a wide range of potential solutions and downsides, and meticulous scrutiny of user personas, among other factors. We struggled with several issues including: (1) disagreements over colours and character designs (eg, some wanted the main characters’ hair to be tied back and others wanted their hair to be untied, respondents gave conflicting critiques about aspects of the characters’ facial features, colour of clothes, etc) and (2) there was a specific scene in which healthcare workers portray dismissive demeanours (that emerged from previous phase^21^), which some healthcare workers themselves wanted removed because they saw it as potentially tarnishing their credibility.

To mitigate this overcrowding, our team engaged with Verganti’s ‘process of innovation of meaning’.^14^ The full process begins with an individual envisioning a challenge, problem or hypothesis, followed by two designers (pairs or sparring partners) undergoing the process of criticisms (ie, attacking each other’s ideas or hypothesis, comparing or combining different ideas) to deepen the solution space. The pairs then assemble into larger groups, called ‘radical circles’, wherein people with divergent opinions and identities are asked to critique the proposed idea, challenge conventional thinking and create innovative solutions.^14^ The term ‘radical’ is used to signify the pursuit of unique views while ‘circle’ denotes the deliberate selection of individuals who collaborate closely, often within the framework of an intense workshop.^14^ Verganti recommends that people build up confidence and gain clarity in their radical circles, and then complete the meaning-making exercise by forming a larger discussion group and engaging in another round of critique and problem-solving.^14^

Verganti’s approach of radical circles appears to run counter to HCD because they foster a whittling of ideas and champion inside-out perspectives^14^; however, we felt that this approach could actually serve as a complement to the criticism wary, outside-in approach (ie, relying solely on feedback and insights from users) of HCD. Consequently, within our HCD process, we strategically included radical circles as a means to elicit more discerning feedback and foster the emergence of novel interpretations. Our approach deviated from the conventional practice of first engaging in pairs, moving to radical circles and subsequently transitioning to bigger discussion groups as part of the comprehensive meaning-making process (see table 1).

**Table 1:** Concepts of Verganti’s process^14^ and our application

Concepts	Objective	Concept description	How we integrated the concept in our study
**Radical circles **refer to selected small group that wants to effect change	To uncover contrasts and tensions within existing design paradigms towards fostering breakthrough solutions	In the typical process of innovation of meaning, the radical circle is responsible for generating a diverse range of ideas, which can vary in terms of their comprehensiveness and level of ambiguity.	In our study, we employed radical circles as an additional technique to address conflicting feedback. We encouraged innovative thinking by providing the radical circles the opportunity to engage in discussions among themselves, specifically through a Zoom call and in-person discussion to explore overcrowded concepts in an inviting environment.
**Interpreters** refer to selected individuals with different expertise to challenge and refine the envisioned intervention	To critically challenge the innovative direction of the design towards generating meaningful designs	The process entails questioning the reasons behind the disintegration of the previous meaning and delving into the narratives of participants in order to expand the concept in novel and innovative ways. When engaging with interpreters, the process entails asking them: ‘What would I love people to love?’ to consider novel interpretations.	Throughout our prototyping phase, our radical circles extended to take on the role of ‘interpreters’ with the objective of going beyond the conventional value and concept of vaccines. Our process then focused on understanding the meaning that our target populations attribute to vaccines and underscore the emotional resonance associated with vaccination. During the prototyping phase, we diligently documented all the criticisms that were raised during the radical circles/interpreters’ sessions via debriefings. We performed critical analysis of our data to further explore the respondents’ perception of the vaccine (‘the what’s: meaning and purpose’) and delved into the reasons behind their decision to receive vaccines (‘the needs’). Subsequently, we devised an envisioning framework (a method of capturing the new meaning by contrasting it to the old one, see figure 1) to aid in the shift towards novel interpretations of the concept of vaccines.
**People** refer to the end-users (receiving and delivering the service), radical circles and interpreters.	To derive new meanings through the process of value creation	When engaging with end-users and radical circles, there is a need to maintain equilibrium between forward-thinking ideas and pragmatic considerations.	We collaborated closely with both end-users and radical circles and maintained regular communication to stay updated on the evolving context within the study settings. We used the Kano methodology in our study to examine how product features influence customer satisfaction.^26 27^ We focused on the attributes of our intervention such as subtitles, content summaries and social media shareability that are associated with enhancing accessibility.^26 27^

While Verganti’s radical circles involve group discussions, due to logistical challenges during COVID-19 pandemic, our radical circles were conducted using a combination of remote FGDs and in-person IDIs. We implemented radical circles with stakeholders who we thought could provide more critical and constructive feedback on our intervention video. We performed in-person IDIs (n=4) with health communication officers with experience in marketing and social media, and one remote FGD (n=3) with health promotion specialists well-versed in print and online resources for building community awareness. During these radical circle sessions, we played the whole video clip silently (to see whether respondents understood the plot) before sharing the full script. The deliberative sessions included revisiting scripts (ie, ensuring that the wordings are clear and free from technical terms), rearranging stories (ie, ensuring that the plot sequence provides flow, builds anticipation and excites the viewers), and rethinking ways to include certain governmental features (ie, use of Philippines Department of Health’s and their ‘Healthy Pilipinas (Healthy Philippines)’ campaign logos) so that the final intervention is aligned with the Philippine Department of Health’s nationwide vaccination and health promotion efforts. Further, from their perspectives, the inclusion of such governmental features provides legitimacy to the final product. Following the radical circle sessions, we further refined the intervention video and began to incorporate intricate details and codevelop the iterated characters of the storyboard.

**Figure 3:**
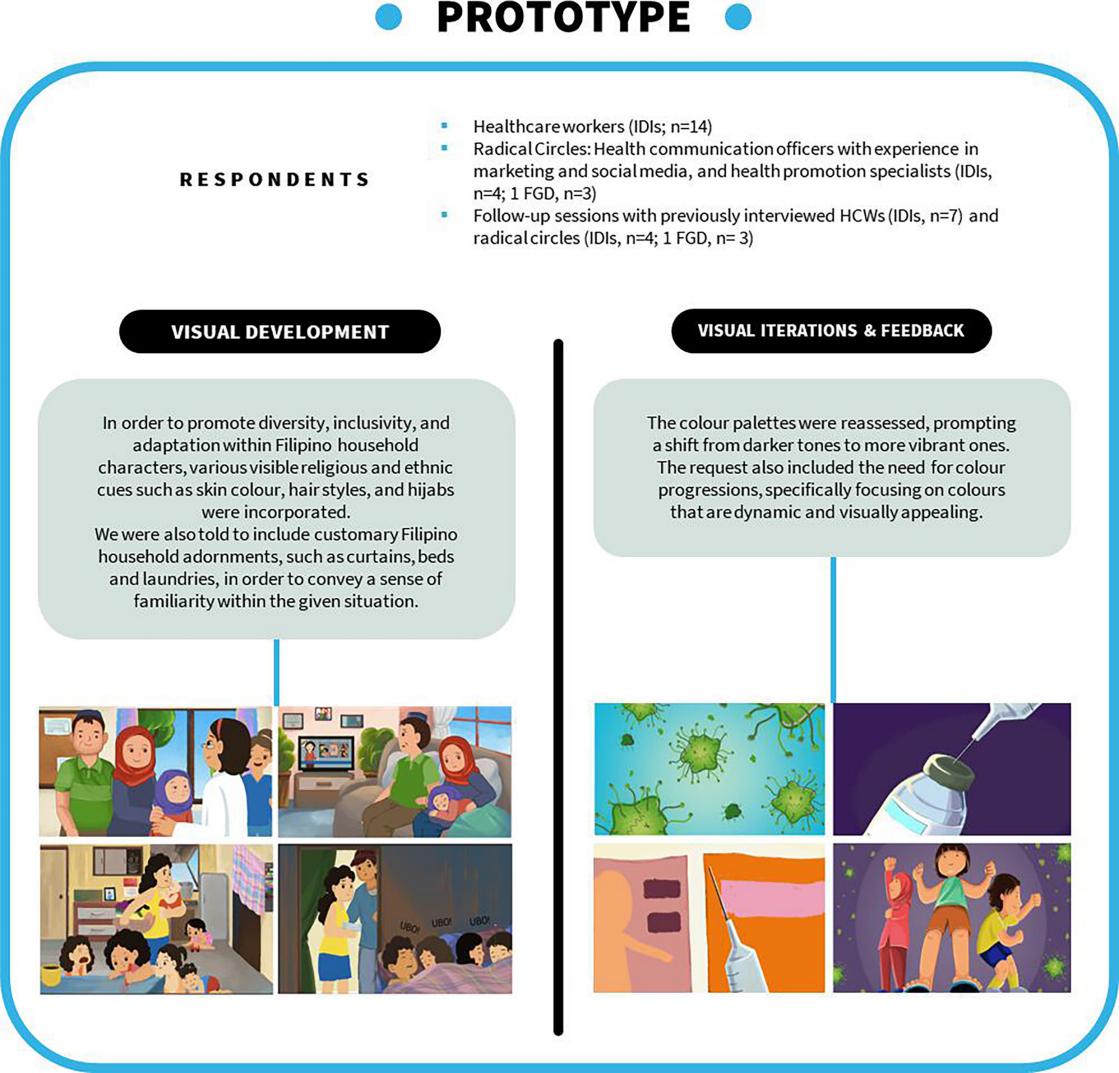
Prototype phase: SALUBONG storyboard progression. FGDs, focus group discussions; HCWs, healthcare workers; IDIs, in-depth interviews.

After incorporating all critical feedback, we returned to our radical circles (1 FGD, n=3; IDIs, n=4) and previously interviewed healthcare workers (follow-up IDIs, n=7) to show again the latest revised version of the video. Here, we redisplayed the video with narrative voice over so respondents could provide more feedback. Colour adjustments (ie, progressive colours from darker tones to glossy and sharp ones), a new voiceover (ie, moving from alarming to a serious to a joyous tone of voice), and video subtitles (ie, having three versions: (1) no subtitles, (2) with English subtitles and (3) with Filipino subtitles) were highlighted as areas for further improvement (see figure 3).

**Figure 4:**
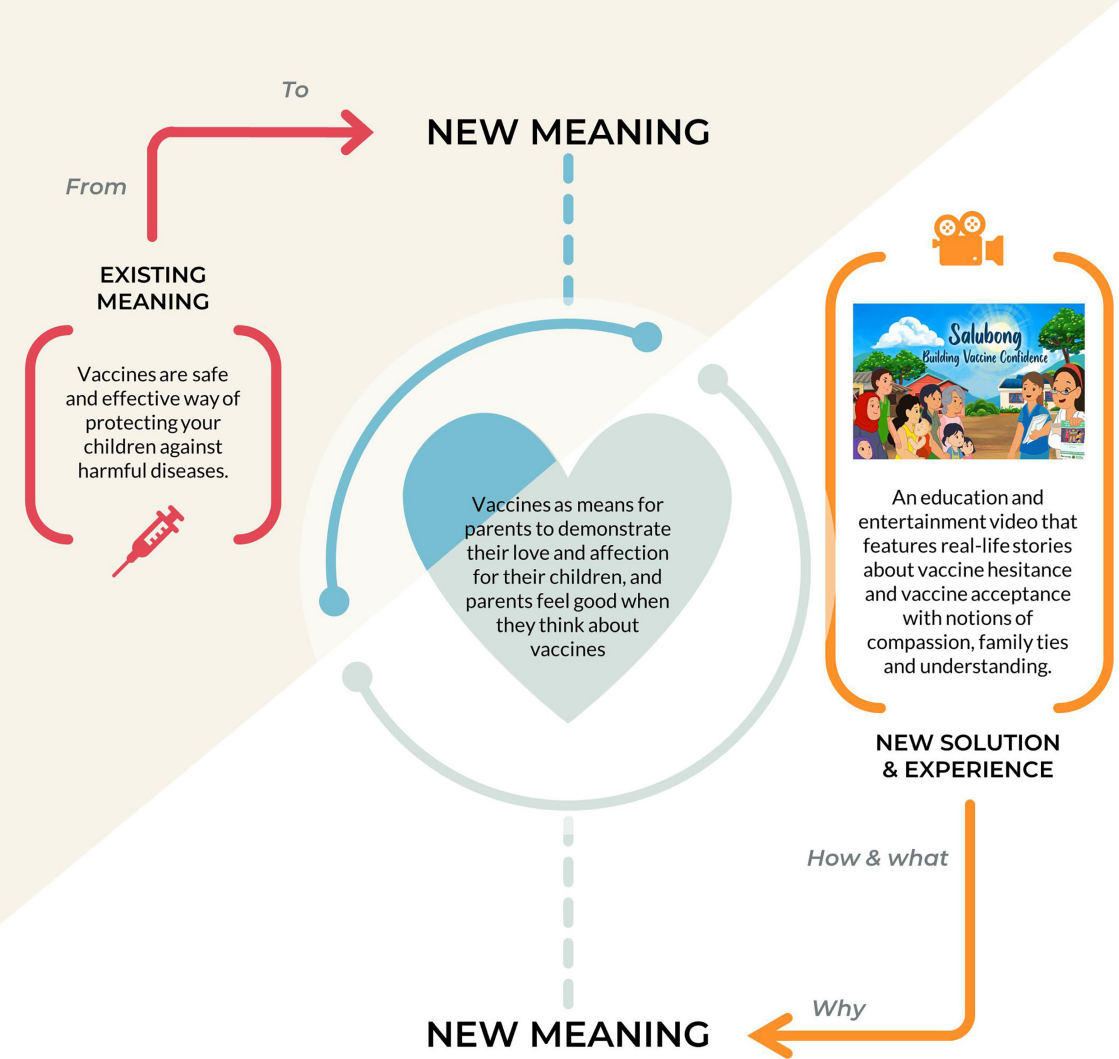
The developed new meaning for the vaccines (‘envisioning framework’ was adapted from Verganti^14^).

Following the radical circles process, our team engaged in debriefing sessions to solidify our intervention’s central tenet and to underscore insights gleaned from the meaning-making approach (see figure 4). Rather than concentrating on the traditional health promotion message of ‘vaccines are safe, free and effective’, we added a new meaning and value to vaccines, which centred on empathic components of love and devotion for children.

At the end of this phase, we were able to finalise our intervention, a five-minute animation ‘Salubong: Building Vaccine Confidence’ that features narratives of Filipino families’ experiences with vaccines (viewable here).

### Step 5: testing

We tested the developed intervention against a control video in a randomised controlled trial involving 719 caregivers of small children.^11^ We incorporated additional questions to our pretest and post-test survey questionnaires in order to further explore the nuanced implications and ascertain the extent to which the emotional impact of our intervention video had been effectively conveyed. We also nested a Kano analysis into the study to categorise intervention quality characteristics and to gauge how these intervention characteristics might link to viewer satisfaction.^26 27^ The Kano results indicated that intervention characteristics related to improving accessibility (subtitles, content summaries, social media shareability) and credibility (institutional logos, trusted messengers, in-person interaction) were the most crucial for viewer satisfaction.^27^

Our HCD-driven intervention proved beneficial in shifting caregivers’ beliefs and expectations towards childhood vaccinations, and respondents who had previously delayed or refused a vaccination for their children reported an increase in vaccine confidence post-intervention.^11^

### Design trade-offs, pivots and lessons learnt

In table 2, we outline challenges and mitigations across the phases of HCD. We recognise that certain difficulties may be method-specific and/or context-specific, but we hope the streamlined bullet points inspire workable solutions.

**Table 2:** Challenges, mitigations and lessons learnt

Challenges within HCD process		How we mitigated these challenges	Lessons learnt
Ethical clearance	Obtaining ethical clearance can be challenging due to the inherent uncertainty around the ultimate product or intervention, which remains unknown until active engagement in the process	We consulted with ethics committees extensively, outlining our methodology in detail (and providing some a priori intervention ideas) to facilitate a better understanding of the nature of our research	Researchers should generate a list of potential intervention ideas for ethical clearance Researchers should plan to engage with the ethics committees on an ongoing basis, including through amendments, if the HCD process suggests products and testing rounds that were not predicted ahead of time
Conflicting preferences among some users	Different population groups have contrasting ideas and perspectives about the problem (in our case, vaccines)	We made more informed decisions about what to include or leave out of our final intervention with the support of ‘radical circles’ (discussions with people who have a substantial understanding of the target population and in negotiating conflict in the development of health promotion materials)	Design teams may be required to make trade-offs between various design objectives, and it is not always easy to reconcile conflicting feedback from various stakeholders Radical circles can challenge the iterated intervention idea, and build a more holistic and unconventional take on the problem When feedback remains vague and conflicting, it may be necessary to incorporate additional techniques to see what works best. Kano survey can help identify features that could be leveraged further or should be removed
Challenge in lessening the empathy gap	Few people were willing to be open and critical while offering their opinions. However, some would say, ‘maganda siya’ (it is beautiful) and ‘okay siya’ (it is okay). Even though most interviewers would follow-up on these types of comments, some nonetheless remain vague	While this may not be unique to HCD, the power of rapport-building and frequent reassurances that no ideas are incorrect or invalid could increase openness	Design teams may facilitate a free-flowing environment where users feel comfortable voicing their thoughts by making the space inviting and safe for everybody Bridge the gap between the researchers and the respondents by facilitating a mutual understanding that the respondent possesses expertise in their own lives and that their insights hold significant value in informing the intervention
Tensions within teams and end-users, multiple views and multiple perspectives, and overcrowding of ideas	Tensions on how many end-users to consult, how long to take, how many iterations to pursue before finalising The narratives might be presented from so many perspectives, making it hard to develop an idea that would resonate with the intended audience Ideas created during brainstorming and ideation sessions are sometimes too numerous to appropriately prioritise. Our design process was slowed by this overcrowding	We performed debriefings and team meetings to understand where profound empathy resulted in significant revelations or advancements. This underscored that empathy is not a barrier, but rather an essential component in achieving effective design solutions We collaborated with a wide range of end-users in radical circles to produce ideas and get constructive and/or radical criticism. This added layer guaranteed that all viewpoints are considered and that the greatest ideas are prioritised We attempted to gather a few excellent ideas rather than accumulating ever larger quantities of ideas. In this way, we curtailed a situation where good ideas got lost and instead focused on those that could spark the most potential to succeed	Design teams need to build in time (and budget) for regular team debriefings where tensions can be identified and discussed. Debriefings need a structured moderation approach so that tensions are dealt productively When there are too many potential solutions, it might be useful to reframe the issue in order to sharpen the emphasis of the design. Narrowing down on the precise nature of the issue at hand might aid in selecting the best solutions
Time-consuming and resource-intensive	Respondents became impatient or exhausted when doing more probing about what they like and do not like, and this impacts the quality of their feedback	We limited the duration of meetings to prevent respondents from feeling stressed. We also asked each respondent whether they were interested in potentially participating in further sessions. Those who agreed were added to a list so we could easily contact potential respondents for next sessions	Design teams need to make sure that feedback systems are effective. Techniques like rapid prototyping and iterative testing may be used for this purpose since they facilitate short feedback loops and reduce the burden placed on participants to offer a large amount of input all at once
Uncertain intervention underpins uncertain testing methods	Since the intervention has to be developed throughout the first phases of HCD, gauging how to assess intervention success is difficult	We used a phase-by-phase evaluation to make revisions to the intervention plan as we went. This method also provides insight into future ethical problems and financial forecasts	After an intervention has been developed and put into action, randomised controlled trial is the preferred method of assessing interventions. Nonetheless, designers should be willing to execute alternate approaches, such as the quasi-experimental design, a common way of causal effect assessment when an experimental design is not possible

### Something radical and something meaningful: moving forward

In our work, the use of radical circles helped us navigate the nuance in feedback and overcrowding of ideas to arrive at a ‘new meaning’, in our case, bolstering vaccine confidence.^10^ Verganti^14^ proposes that radical circles should be a distinct and novel process to invigorate the design thinking approaches and challenge the ‘sea of sameness’ of goods and services due to the widespread adoption of an HCD approach. Verganti argues that designers should take a more radical approach based on exploring and developing novel meanings.^14^ Rather than concentrating on how issues may be addressed, we should instead concentrate on the meanings and on why end-users would enjoy our products or interventions.^14^ To do this, one must use creative, out-of-the-box thinking to come up with novel, useful ideas, rather than depending primarily on people’s preferences as a starting point.

When we became overwhelmed amid our design process, we drew on Verganti’s recommendations as a means to break through. Radical circles provided a space for those who held opposing or critical viewpoints about vaccines to discuss and share in a diffusing environment. The acceptance of tension broadened our design process; we devised an intervention that gently critiqued the rigidity and lack of human care within the health system, but we did this in a manner that was attuned to Filipino perspectives and respectful of challenges healthcare workers themselves face. HCD and the use of radical circles revealed that people desired messages framed around love and responsibility for one’s children with honest discussion of vaccine side effects.^10^ These approaches enabled us to create credible, resonant stories and characters to deliver these messages potentially contributing to the ultimate success of the final intervention in pilot-testing.^11^

While we ultimately decided for employing radical circles in our own work, there are several underexplored practices that could show promise in addressing the challenges of HCD. We encourage global health scholars, intervention designers and implementors to explore different techniques such as design charettes, speed dating and world cafés, among others^24^ that might facilitate the collaborative generation of meaningful ideas. Design thinking tools, such as mind maps (www.xmind.com), affinity diagrams (www.lucidchart.com), impact and feasibility matrices, and the use of miro software (www.miro.com), can be advantageous for the organisation and prioritisation of ideas.

## Conclusions

HCD is complex, messy and time-consuming but creates a design space where end-users’ demands and experiences are acknowledged and prioritised. Radical circles can assist in managing the complexities encountered and can reimagine meanings. It is crucial, however, to be aware of the limitations of HCD and to make concerted efforts to overcome them by including many viewpoints, expanding the scope of the questions and striking a balance between usability and other considerations.

## Supplementary material

10.1136/bmjgh-2023-014870 online supplemental file 1

## Data Availability

All data relevant to the study are included in the article or uploaded as online supplemental information.
