# Supplementary material for: Messy but worth it: human-centred design as applied within a successful vaccine-promotive campaign
Source: BMJ Glob Health. 2024 Jul 29;9(7):e014870. doi: 10.1136/bmjgh-2023-014870 (PMC11288141; doi:10.1136/bmjgh-2023-014870)
Supplement: online supplemental file 1 [file bmjgh-9-7-s001.pdf]

Supplemental Material 1

Author Reflexivity Statement

1. How does this study address local research and policy priorities?

In the aftermath of the Dengvaxia controversy, vaccine hesitancy was identified as a research and policy priority in the Philippines by the Philippines Department of Health (DOH). The Research Institute for Tropical Medicine (RITM), the research arm of the DOH and a partner in this study, has since been working on novel approaches to regain trust in vaccination and health programs. The topic of this study therefore directly aligns with local research and policy priorities.

2. How were local researchers involved in study design?

The overarching research project was developed in partnership between RITM and the Heidelberg Institute of Global Health (HIGH), Germany. MDCR approached SAM with the idea to work on vaccine hesitancy in the Philippines, and research team members from RITM and HIGH then jointly developed the study plan. Study procedures were repeatedly adapted to respond to changing circumstances in the Philippines (e.g., with regards to COVID-19 pandemic-related travel restrictions) based on the expertise of RITM researchers.

3. How has funding been used to support the local research team?

Of the original funding for this research project (100,000 USD), approximately 50% were disseminated directly to RITM partners with a majority of funds being used for in-country data collection, the local design of study and intervention materials, etc. All funding allocation decisions, both when preparing the original budget and when considering funds reallocation, were made jointly by RITM and HIGH study leads. Additionally, RITM researchers repeatedly drew on open access publication support offered by the Bill and Melinda Gates Foundation. This project has also led to follow-up funding for the local research team, including a scholarship for MDCR to pursue their PhD (ca. 40,000 USD) and RITM support for follow-up research activities in the same field for JL and VE (ca. 6,000 USD each).

4. How are research staff who conducted data collection acknowledged?

All research staff who conducted data collection were invited and included as co-authors in this article.

5. Do all members of the research partnership have access to study data?

All members of the research partnership have access to study data.

6. **How was data used to develop analytical skills within the partnership?**

For the purpose of this article, MDCR and SAM collaborated closely as they developed the analytical approach. Building analytical capacity among all partners has been a cornerstone of the ongoing partnership, with several local researchers leading their first own data analysis and publication. Several further capacity building activities were conducted, covering both qualitative and quantitative methods.

7. **How have research partners collaborated in interpreting study data?**

Data were interpreted in close collaboration between international and local partners. Insights from this analysis and how they could inform other research activities were routinely discussed between members of the research team.

8. **How were research partners supported to develop writing skills?**

As part of the overarching partnership, SAM has provided in-depth writing guidance to study team members which, as of now, led to five local researchers leading their first scientific publication based on data from this collaboration. Additionally, an in-depth workshop on the write-up and dissemination of scientific findings, led by SAM, MDCR, KB and KR, were held in summer 2023 at RITM.

9. **How will research products be shared to address local needs?**

All publications coming from this partnership, including the article at hand, were open access. We are also routinely sharing results from this partnership via policy briefs and stakeholder meetings with key local bodies, utilizing established RITM and DOH channels.

10. **How is the leadership, contribution and ownership of this work by LMIC researchers recognised within the authorship?**

All local researchers who have contributed to this study are included as co-authors or acknowledged by name (see section 4). The majority of co-authors are from the Philippines (VE, JL, MA, JLG, TAB), including the lead author (MDCR). KB, JW, KR, TB, and SAM are the HIC co-authors of this article.

11. **How have early career researchers across the partnership been included within the authorship team?**

KB, KR, TB, and SAM are the only co-authors who have already completed their PhDs. MDCR (who has already obtained a doctorate in nursing) and JW are currently pursuing their PhD, and several other co-authors are in the process of or planning to obtain graduate training in Global Health or related fields.

12. How has gender balance been addressed within the authorship?

The author team includes non-binary (1), male (2), and female (8) individuals. We sought to mitigate the gender imbalance originating from most co-authors identifying as female by encouraging constant and open communication in routine team meetings and throughout all study processes.

13. How has the project contributed to training of LMIC researchers?

As outlined in section 11, most co-authors are in their pre-doctoral phase and in-depth training on data collection, analysis and writeup is integrated into all stages of the project. As a result, several local research team members have published first-authored articles or are currently developing such articles.

14. How has the project contributed to improvements in local infrastructure?

This project has not directly contributed to infrastructural improvements in the Philippines or in Germany.

15. What safeguarding procedures were used to protect local study participants and researchers?

All study procedures were approved by the institutional review boards at HIGH and RITM and followed the Philippines' National Ethical Guidelines for Health and Health Related Research, as well as the tenets of the declaration of Helsinki and the Belmont report. To protect participants and researchers, data collection was shifted online in the context of the COVID-19 pandemic, and all data was pseudonymized in line with the Philippines Republic Act 10173 (Data Privacy Act). Local study team members could voice emerging concerns in regular team meetings where strategies for alleviating such concerns were discussed and implemented.
